# Association between *P16^INK4a^* Promoter Methylation and Ovarian Cancer: A Meta-Analysis of 12 Published Studies

**DOI:** 10.1371/journal.pone.0163257

**Published:** 2016-09-20

**Authors:** Xiyue Xiao, Fucheng Cai, Xun Niu, Hao Shi, Yi Zhong

**Affiliations:** 1 Department of Obstetrics and Gynecology, Union Hospital, Tongji Medical College, Huazhong University of Science and Technology, Wuhan, China; 2 Department of Pediatrics, Union Hospital, Tongji Medical College, Huazhong University of Science and Technology, Wuhan, China; 3 Department of Otorhinolaryngology, Union Hospital, Tongji Medical College, Huazhong University of Science and Technology, Wuhan, China; 4 Department of Epidemiology and Biostatistics, and the Ministry of Education Key Lab of Environment and Health, School of Public Health, Tongji Medical College, Huazhong University of Science and Technology, Wuhan, China; Van Andel Institute, UNITED STATES

## Abstract

**Background:**

Ovarian cancer is the primary cause of death in women diagnosed with gynecological malignancies worldwide. Absence of early symptoms prevents prompt diagnosis or successful therapeutic intervention. *P16*^*INK4a*^ is a well-known tumor suppressor gene (TSG). Aberrant methylation of TSG promoter is an important epigenetic silencing mechanism leading to ovarian cancer progression. Studies have reported differences in methylation frequencies of the *p16*^*INK4a*^ promoter between ovarian cancer and the corresponding control group. However, the association between *p16*^*INK4a*^ promoter methylation and ovarian cancer remains unclear and controversial. Therefore, a meta-analysis was conducted to clarify the relationship between *p16*^*INK4a*^ promoter methylation and ovarian cancer.

**Methods:**

PubMed, Web of Science, EMBASE and CNKI were searched to identify eligible studies for the evaluation of the association between *p16*^*INK4a*^ promoter methylation and ovarian cancer. Odds ratio (ORs) and 95% confidence intervals (95%CI) were calculated to determine the strength of association between *p16*^*INK4a*^ promoter methylation and ovarian cancer.

**Results:**

A total of 612 ovarian cancer patients and 289 controls from 12 eligible studies were included in the meta-analysis. Overall, a significant association was observed between *p16*^*INK4a*^ methylation status and ovarian cancer risk using a fixed-effects model (OR = 2.02, 95% CI = 1.39–2.94).

**Conclusion:**

The results of our meta-analysis show that aberrant methylation of *p16*^*INK4a*^ promoter was significantly associated with ovarian cancer. It may represent a promising molecular marker to monitor the disease and provides new insights into the treatment of human ovarian cancer.

## Introduction

Ovarian cancer is the primary cause of death in women with gynecological malignancies. Based on GLOBOCAN estimates, more than 238, 700 new cancer cases were diagnosed and nearly 151,900 died from ovarian cancer worldwide in 2012 [1]. Absence of early symptoms prevents prompt detection or therapy of ovarian cancer. Approximately 75% of ovarian cancers are diagnosed at an advanced stage [2,3]. Therefore, early diagnosis and prevention depend on the ability to identify genetic and epigenetic events underlying the initiation and progression of the disease. Recent advances in molecular oncology have facilitated the identification and understanding of several genetic and epigenetic events that contribute to ovarian carcinogenesis [4–7].

Molecular genetic alterations, including activation of proto-oncogenes and inactivation of tumor suppressor genes (TSG), may play a key role in tumorigenesis. Epigenetic inactivation of genes following methylation of CpG islands in promoters is one of the most frequent events encountered in human tumors. The tumor suppressor gene *p16*^*INK4a*^ is a major target for carcinogenesis in various human tumors [8–11]. It is a negative regulator of cell cycle. *P16*^*INK4a*^ prevents the inactivation of retinoblastoma (Rb) protein by inhibiting cyclin-dependent kinases (CDks). Retinoblastoma (Rb) protein has diverse tumor-suppressor functions and plays an important role in apoptosis and cell cycle regulation [12]. Studies have shown that methylation of *p16*^*INK4a*^ promoter may play a critical role in the development of ovarian cancer.

Until now, a few studies reported the differences in methylation frequencies of *p16*^*INK4a*^ promoter between ovarian cancer and non-cancerous tissues. However, these findings are inconsistent. Therefore, the objectives of this meta-analysis are to consolidate the available data and to clarify the association between *p16*^*INK4a*^ promoter methylation and human ovarian cancer.

## Materials and Methods

The meta-analysis was performed according to the latest Preferred Reporting Items for Systematic Reviews and Meta-Analyses (PRISMA).

### Search Strategy

Relevant studies were identified from online electronic databases (PubMed, Web of Science, EMBASE and CNKI) using the following key words: (ovarian OR ovary) AND (cancer OR carcinoma OR tumor) AND (*p16* methylation). Articles were retrieved up to May 3, 2016.

### Study Selection

Three independent reviewers (Xiyue Xiao, Yi Zhong, and Fucheng Cai) screened the titles and abstracts retrieved in the electronic search to identify relevant studies. The inclusion criteria were: (1) case-control study design, (2) data necessary for calculating odds ratios (ORs), (3) studies primarily evaluating the association between *p16*^*INK4a*^ methylation and ovarian cancer, (4) incidence of *p16*^*INK4a*^ methylation in both case and control groups, and (5) sample types limited to tissues. According to the inclusion criteria, the title and abstracts from the preliminary search were evaluated. All potentially relevant articles were evaluated in full. Abstracts, letters to the editor and case reports were not included. Finally, a total of 12 articles [13–24] were included in our meta-analysis involving 612 cases and 289 controls.

### Data Extraction and Quality Assessment

The selected studies were reviewed by three independent reviewers (Xiyue Xiao, Yi Zhong, and Fucheng Cai). The following information was extracted from the eligible studies: first author’s name, year of publication, study population, the number of people with *p16*^*INK4a*^ methylation in the case and control groups, the number of case and control groups, the measurement methods of methylation and control types. All the data in the included studies were checked by two reviewers (Xun Niu and Hao Shi) as described in the Cochrane Handbook for systematic reviews.

### Statistical Analysis

The pooled odds ratios (ORs) and their 95% confidence intervals (CIs) were calculated to assess the strength of the association between *p16*^*INK4a*^ methylation and ovarian cancer. Statistical heterogeneity was analyzed on the basis of *I*-square (*I*^*2*^) value. An *I*^*2*^ value above 75% indicated high heterogeneity, an *I*^*2*^ value between 50% and 75% suggested moderate heterogeneity, and an *I*^*2*^ value between 25% and 50% indicated low heterogeneity. A result was homogeneous when the *I*^*2*^ value was less than 25%. If *I*^*2*^< 50%, the studies were considered homogeneous or low in heterogeneity. A fixed-effects model was used to combine the effect size. If *I*^*2*^> 50%, the studies were believed to be moderately or highly heterogeneous, and the random-effects model was used to combine the effect size [25,26]. A subgroup analysis was conducted to assess the impact of race (Asia and Caucasus), method (BSP and MSP), and control type (blood and tissues). Potential publication bias was assessed by Funnel plot [27], Begg’s test, and Egger’s test [27,28]. According to the sensitivity analysis, the contribution of each study to the final results of the meta-analysis was evaluated. All the *p* values were two sided with a significant level at 0.05. All statistical analyses were performed with the Meta package (version 2.2–1) in R (version 3.00).

## Results

### Search Results

**Fig 1:**
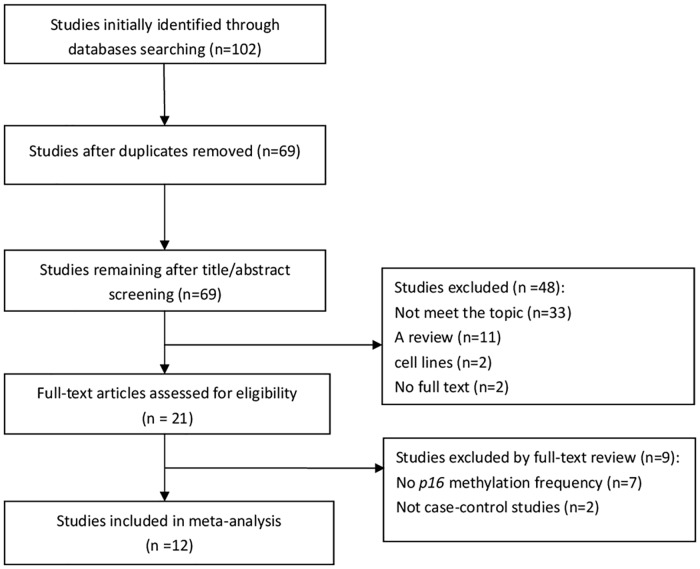
Flowchart outlining study selection in the meta-analysis.

The initial search was independently executed by three reviewers (Xiyue Xiao, Yi Zhong, and Fucheng Cai), and 69 articles were initially selected. The 69 articles were quickly screened by abstract and title based on inclusion/exclusion criteria. After careful review by the three experts, 21 articles were found to be related toour meta-analysis. These articles underwent a second review. Finally, a total of 12 studies were included in the meta-analysis. The detailed steps involved in the literature search are shown in Fig 1.

### Study Characteristics

Twelve studies comprising data from a total of 612 cases and 289 controls were included in this review. Eight of these studies involved Asian subjects, and four studies investigated Caucasians. Among the included studies investigating *p16*^*INK4a*^ methylation in ovarian cancer and controls, three utilized bisulfite sequencing PCR (BSP) and nine employed methylation-specific polymerase chain reaction (MSP). The control group included peripheral blood and tissues (benign ovarian tissues, normal ovarian tissues of cancer-free patients and adjacent tissues). Characteristics of the 12 studies are summarized in Table 1.

**Table 1:** Characteristics of included studies.

Author	Year	Country	Age(y)	Case		Control		Method	Case type	Control type
				M	U	M	U			
Bammidi LS	2012	India	20–75	28	22	18	32	BSP	tissues	blood
Bhagat R	2014	India	20–79	58	76	11	30	MSP	tissues	tissues
Li M	2006	China	NA	6	12	0	10	MSP	tissues	tissues
Liu Z	2005	USA	27–81	13	39	15	25	MSP	tissues	tissues
Makarla P	2005	USA	51.5(20–86)	7	16	3	36	MSP	tissues	tissues
Niederacher D	1999	Germany	NA	6	17	0	10	MSP	tissues	tissues
Shih YC	1997	Australia	NA	0	45	0	2	MSP	tissues	tissues
Tam KF	2007	China	53.1±1.4	17	72	5	30	MSP	tissues	tissues
Wong YF	1999	China	NA	2	47	0	10	MSP	tissues	blood
Shen W	2008	China	52.2, 53.2	13	50	0	30	BSP	tissues	tissues
Wei W	1999	China	46.5±12.47	5	21	0	2	BSP	tissues	tissues
Xu B	2003	China	NA	4	36	0	20	MSP	tissues	tissues

Blood: peripheral blood samples from each patient with ovarian cancer; tissues: benign ovarian tissues, normal ovarian tissues of cancer-free patients and adjacent tissues; MSP: methylation-specific polymerase chain reaction; BSP: bisulfite sequencing PCR; NA: not available; M: methylation; U: unmethylation.

### Combined Analysis of Included Studies

**Fig 2:**
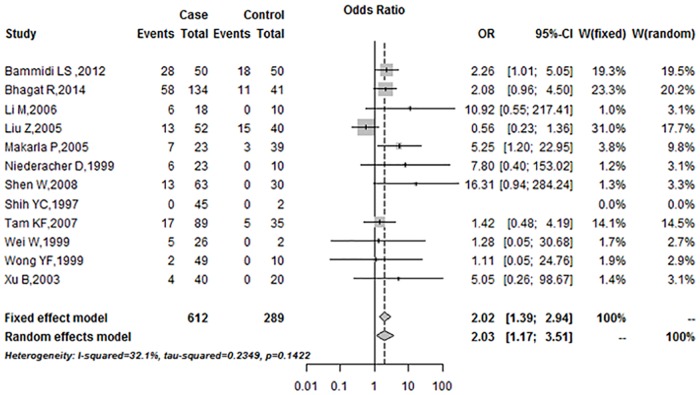
Summary estimates for *p16*^*INK4a*^ promoter methylation frequency associated with ovarian cancer.

The combined analysis showed the relationship between *p16*^*INK4a*^ promoter methylation and ovarian cancer risk (Fig 2). A fixed-effects model was employed due to low heterogeneity among the included studies (*I*^*2*^ = 32.1%). In the overall meta-analysis, *p16*^*INK4a*^ promoter methylation frequency was significantly associated with ovarian cancer (Summary OR was 2.02, 95%CI = 1.39–2.94).

### Sensitivity Analysis

**Fig 3:**
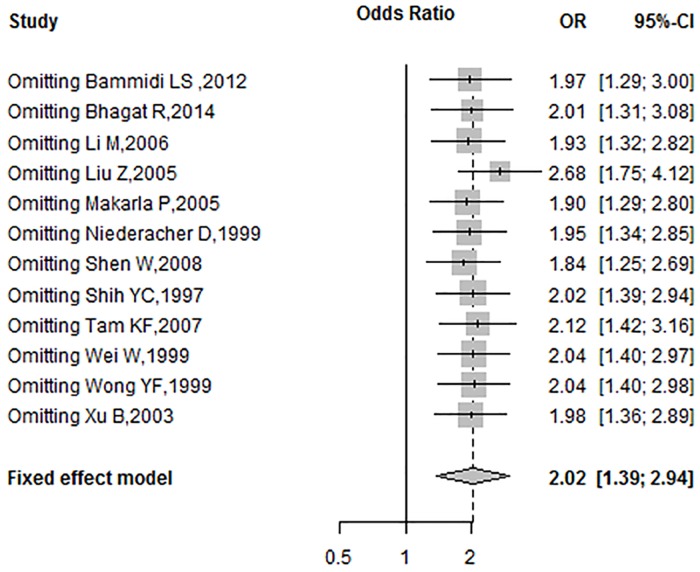
Sensitivity analysis of pooled OR for *p16*^*INK4a*^ methylation and ovarian cancer under the fixed-effects model.

A sensitivity analysis was performed by omitting a single study and calculating the pooled OR for the remaining studies under the fixed-effects model, to determine the effects of each individual study. The results of sensitivity analysis are summarized in Fig 3. According to sensitivity analysis, the OR ranged from 1.84 (95%CI = 1.25–2.69) to 2.68 (95%CI = 1.75–4.12) by omitting a single study in the fixed-effects model. The pooled OR values between *p16*^*INK4a*^ promoter methylation and ovarian cancer were reliable and stable.

### Subgroup Analysis

**Fig 4:**
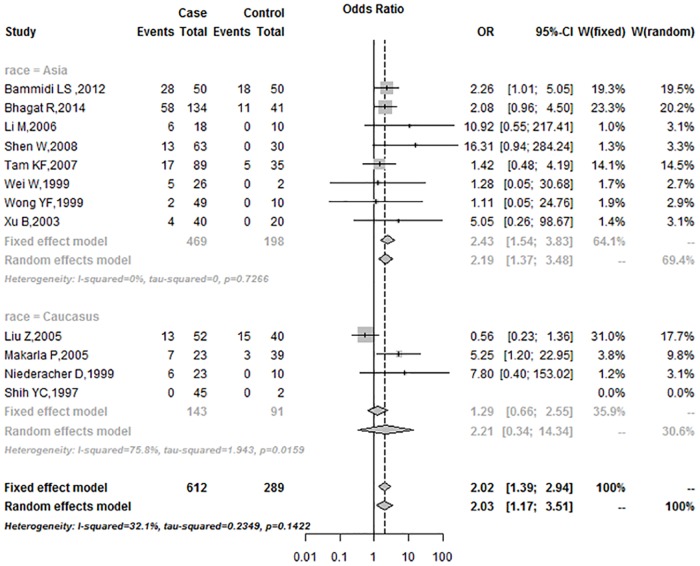
Subgroup analysis based on race.

**Fig 5:**
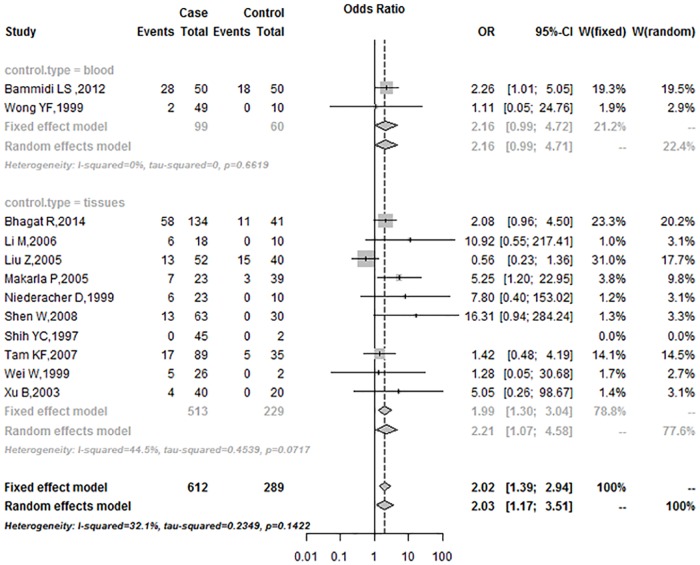
Subgroup analysis based on control sample type.

**Fig 6:**
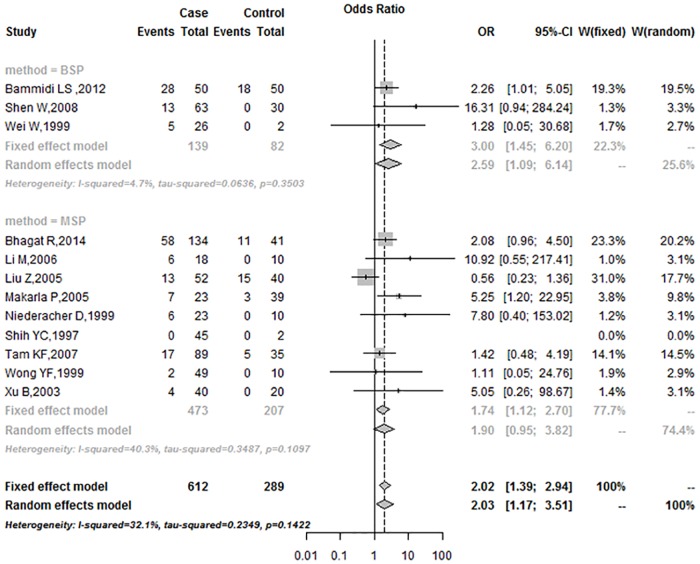
Subgroup analysis based on methylation detection method.

In the subgroup analysis based on race, the OR was 2.43 (95%CI = 1.54–3.83) in Asians, and 2.21 (95%CI = 0.34–14.34) in Caucasians (Fig 4). Subgroup analysis of the control sample showed that the frequencies of *p16*^*INK4a*^ promoter methylation in the blood group were higher than in the tissue group under the fixed-effects model (Blood: 2.16, 95%CI = 0.99–4.72; Tissues: 1.99, 95%CI = 1.30–3.04; respectively) (Fig 5). According to the mode of methylation detection for *p16*^*INK4a*^ promoter, the OR was 3.00 (95%CI = 1.45–6.20) in the BSP group and 1.74 (95%CI = 1.12–2.70) in the MSP group, under the fixed-effects model (Fig 6).

### Publication Bias

**Fig 7:**
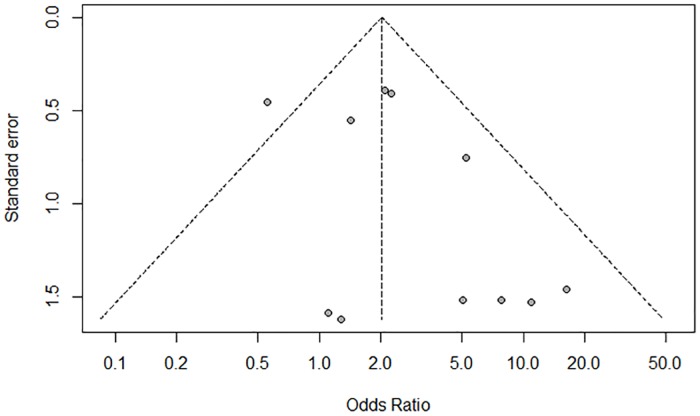
Funnel plot showing minor publication bias.

Funnel plot, Begg’s test, and Egger’s test were used to evaluate the publication bias of the studies. The funnel plot was not perfectly symmetrical (Fig 7), suggesting a slight publication bias. However, the Begg’s(P = 0.59) and Egger’s tests (P = 0.15) showed no evidence of publication bias in our meta-analysis. In addition, the ‘trim and fill’ method showed that no study required statistical correction for funnel plot asymmetry.

## Discussion

The pathogenesis of human ovarian cancer involves complex, multistep and multi-factorial mechanisms including a variety of genetic and epigenetic abnormalities, signal transduction pathways, apoptosis, angiogenesis, and cell cycle regulation. Epigenetic inactivation of the tumor suppressor gene (*TSG*) following promoter methylation of CpG islands is one of the most frequent events in human tumors. Several studies suggest that inactivation of *p16*^*INK4a*^ induced by aberrant hypermethylation may play an important role in the carcinogenesis of lung, liver, stomach, breast, and uterus [29–33]. In a recent meta-analysis from six eligible studies, including 261 patients, Hu et al found that *p16*^*INK4a*^ promoter hypermethylation is correlated with an increased risk of endometrial carcinoma [33]. Similarly, our earlier meta-analysis reported a significant hypermethylation of *p16*^*INK4a*^ promoter in head and neck squamous cell carcinoma (HNSCC) [34].

The current meta-analysis investigated the association between *p16*^*INK4a*^ promoter methylation and human ovarian cancer. It included 12 studies comprising 612 cases and 289 controls. The pooled OR under a fixed-effects model was 2.02 (95%CI = 1.39–2.94) in the cancer cases compared with the controls. The result showed that methylation of *p16*^*INK4a*^ promoter led to a 2.02-fold increased risk of human ovarian cancer compared with the control group. Begg’s tests (P = 0.59) and Egger’s tests (P = 0.15) revealed no publication bias in this study. The sensitivity analysis showed that exclusion of any single study did not affect the overall results or conclusions. Therefore, the results of our meta-analysis are reliable and show relatively strong statistical power.

In the subgroup analysis, the OR for was 2.43 (95%CI = 1.54–3.83) in Asians under the fixed-effects model, and 2.21 (95%CI = 0.34–14.34) in Caucasians under the random-effects model. The association between *p16*^*INK4a*^ promoter methylation and human ovarian cancer in Asians was stronger than in Caucasians. The findings may be attributed in large part to a combination of differences in allele frequencies and complex epistasis or gene-environment interactions [35]. In subgroup analysis, the OR was 3.00 (95%CI = 1.45–6.20) in the BSP group under the fixed-effects model, and 1.74 (95%CI = 1.12–2.70) in the MSP group under the fixed-effects model, respectively. In the past few years, several methods were developed to detect aberrant gene methylation (e.g., BSP, MSP, QMSP, and Pyro). MSP is a simple, sensitive, and specific method for detection of methylation status in CpG-rich regions [36]. However, MSP requires specific gene sequence data for the design of PCR primers, and different primers may influence the results of methylation analyses. In addition, MSP (a non-quantitative non-fluorometric PCR method) failed to detect low levels. BSP provides a more direct and quantitative analysis of most CPG sites within a defined region than MSP [37]. In our study, the data from both BSP and MSP methods showed that the frequency of promoter methylation in *p16*^*INK4a*^ was higher in ovarian cancer than in control. Subgroup analysis of the control sample type showed that the OR was 2.16 (95%CI = 0.99–4.72) in the blood group, and 1.99 (95%CI = 1.30–3.04) in the tissues, respectively, under the fixed-effects model. Subgroup analysis revealed that aberrant methylation of *p*^*16INK4a*^ promoter was significantly associated with ovarian cancer, regardless of race, control sample type and detection method.

However, potential limitations need to be considered when interpreting the results of our meta-analysis. First, since the 12 included studies were retrospective, a potential unidentified confounding, information and selection bias may exist. Second, the population size of the studies included was relatively small. Further investigations with large sample sizes are required. Additionally, we did not explore the association between *p16*^*INK4a*^ promoter methylation and disease characteristics (stage, metastasis, relapse and so on) in human ovarian cancer. The association between *p16*^*INK4a*^ promoter methylation and disease characteristics may highlight the unique role of *p16*^*INK4a*^ promoter methylation in human ovarian cancer.

In conclusion, the results demonstrate that aberrant methylation of *p16*^*INK4a*^ promoter was associated with human ovarian cancer, suggesting that promoter methylation of *p16*^*INK4a*^ plays a crucial role in human ovarian cancer. It may serve as a potential biomarker for early detection and diagnosis of human ovarian cancer.

## Supporting Information

S1 Checklist PRISMA checklist. (DOC)

S2 Checklist Meta-analysis on genetic association studies checklist. (DOCX)
